# Color and Luminance Separated Enhancement for Low-Light Images with Brightness Guidance

**DOI:** 10.3390/s24092711

**Published:** 2024-04-24

**Authors:** Feng Zhang, Xinran Liu, Changxin Gao, Nong Sang

**Affiliations:** Key Laboratory of Image Processing and Intelligent Control, School of Artificial Intelligence and Automation, Huazhong University of Science and Technology, Wuhan 430074, China; fengzhangaia@hust.edu.cn (F.Z.); xinran_liu@hust.edu.cn (X.L.); nsang@hust.edu.cn (N.S.)

**Keywords:** low-light image enhancement, diffusion models, Retinex decomposition strategy, brightness guidance

## Abstract

Existing retinex-based low-light image enhancement strategies focus heavily on crafting complex networks for Retinex decomposition but often result in imprecise estimations. To overcome the limitations of previous methods, we introduce a straightforward yet effective strategy for Retinex decomposition, dividing images into colormaps and graymaps as new estimations for reflectance and illumination maps. The enhancement of these maps is separately conducted using a diffusion model for improved restoration. Furthermore, we address the dual challenge of perturbation removal and brightness adjustment in illumination maps by incorporating brightness guidance. This guidance aids in precisely adjusting the brightness while eliminating disturbances, ensuring a more effective enhancement process. Extensive quantitative and qualitative experimental analyses demonstrate that our proposed method improves the performance by approximately 4.4% on the LOL dataset compared to other state-of-the-art diffusion-based methods, while also validating the model’s generalizability across multiple real-world datasets.

## 1. Introduction

Low-light enhancement is a crucial yet challenging aspect of image processing. Under low-light conditions, images are deteriorated by reduced brightness and a poor signal-to-noise ratio (SNR), obscuring details and compromising the quality. This issue, primarily due to sensor limitations in capturing weak light, results in noise that degrades the image and can be intensified during enhancement, causing color distortions. Therefore, it is crucial to develop methods to improve the visibility and quality.

Various convolutional neural networks (CNNs) [1,2,3,4,5,6,7,8] have been proposed in the field of low-light image enhancement, many of which are based on Retinex theory [9]. This theory guides the separation of images into reflectance and illumination maps. Diverse methods [10,11,12] leverage Transformer models to restore the decomposed maps. However, these approaches cannot be developed further due to the limited representation capabilities of CNN-based models.

Recently, diffusion models have become increasingly important in image enhancement tasks [13], including low-light enhancement [14,15,16,17,18,19,20], due to their excellent performance in modeling complex noise and artifact distributions in images. Ref. [14] employs early downsampling and a global corrector to accelerate processing and mitigate color shifts. Ref. [16] also uses downsampling and gradual upsampling, but with a unique normalization strategy. Ref. [20] incorporates prior knowledge, using color maps to represent image color information. Refs. [15,17] introduce novel components; Ref. [15] reduces the input size through a wavelet transform and maintains details with a high-frequency restoration module, while [17] enhances the effectiveness by feeding degradation features from a designed DGNET to a U-Net in the diffusion model. However, these methods are susceptible to color shifts, making the design of complex modules for color adjustment necessary.

Alternatively, some works [18,19] attempt to combine the diffusion model with Retinex theory, which mitigates the issue of color shifts. Ref. [18] uses a Transformer-based structure for decomposition and conditional diffusion models to restore reflectance and illumination maps. Ref. [19] follows a similar approach but improves the decomposition method and includes a refined module for enhanced color and detail restoration. Ref. [21], on the other hand, uses the diffusion model to extract auxiliary features during Retinex decomposition, integrating these into the RGformer network for enhancement. Ref. [20] indicates that using low-light images and normal-light images with mixed noise directly as inputs to conditional diffusion models yields subpar results, leading to the introduction of additional prior knowledge like color maps and illumination embedding. However, these methods utilize learning-based networks to predict estimates of the reflectance and illumination maps, and such methods are inaccurate while causing the loss of some image structure information.

In this paper, we present a simple but effective image decomposition strategy as a unique paradigm for Retinex decomposition. Specifically, we utilize the grayscaled image as the illumination map; then, we divide the RGB channels of the input image pixel by pixel by the grayscaled image to obtain the reflectance map, and we enhance the reflectance and illumination maps separately using a diffusion model. Furthermore, we introduce brightness guidance to guide the brightness adjustment module to better learn the illumination pixel distribution of the reference images. Extensive experiments demonstrate that our model performs favorably against existing state-of-the-art methods.

The main contributions of this work are as follows:

- We propose a simple but effective image decomposition strategy, which can serve as a unique paradigm for Retinex decomposition;
- We introduce brightness guidance to guide the brightness adjustment and remove the disturbance of the diffusion model;
- We conduct extensive experiments on a benchmark dataset to demonstrate the feasibility of our proposed method.

## 2. Related Work

### 2.1. Traditional Methods

Traditional low-light image enhancement methods can be broadly categorized into three types: gamma correction (GC) [22], histogram equalization (HE) [23], and Retinex theory [9]. Bennet et al. [24] proposed employing bilateral filtering to decompose low-light observations, followed by applying gamma curve methods with different parameter settings to process the decomposed layers and then recombining them. Additionally, Yuan et al. [25] attempted to perform gamma curve operations on each sub-region generated through segmentation. Furthermore, Rahman et al. [26] introduced an adaptive gamma correction method, which dynamically determines the intensity transformation function based on the statistical characteristics of the image. Overall, the existing gamma correction-based methods still suffer from the largest problem of uneven exposure phenomena. Pizer et al. [27] proposed adaptive histogram equalization (AHE) to map the histograms of local regions to simple mathematical distributions. Building upon the principles of human visual locality, Pizer et al. [23] further introduced contrast-limited adaptive histogram equalization (CLAHE). Jobson et al. [28] made some initial attempts based on Retinex theory, estimating illumination through filter introduction, but obtained results that deviated from the distribution of real natural images, leading to unknown artifacts and color biases. With deeper exploration, a series of works [29,30,31] have focused on designing regularization terms for variables within the Retinex model to assist in estimating accurate target variables.

### 2.2. Homomorphic-Based Methods

Homomorphic filtering is a technique that operates in the frequency domain to separate illumination and reflectance components in images. This method is particularly advantageous in enhancing low-light images, where non-uniform lighting conditions prevail. By transforming the multiplicative relationships of these components into additive ones via a logarithmic domain, homomorphic filtering allows for the differential manipulation of illumination and reflectance, thus enhancing the visibility and details in dark regions while preserving the quality of well-lit sections. Sobbahi et al. [32] present a novel approach by embedding homomorphic filtering within a deep learning architecture. The model not only enhances low-light images but also tailors the enhancement process to improve subsequent image classification tasks. This integration demonstrates the dual benefit of image preprocessing for visual improvements and performance boosts in machine learning applications. Chavarín et al. [33] integrate cluster-chaotic optimization with homomorphic filtering. The chaotic optimization approach adjusts the filter parameters dynamically, optimizing the contrast enhancement process. The adaptation is guided by the peculiarities of the image content, leading to superior enhancement results compared to static parameter selection. While effective, homomorphic-based approaches face challenges such as noise sensitivity, computational complexity, parameter sensitivity, poor handling of non-uniform illumination, complex integration with advanced models, and reliance on specific illumination models.

### 2.3. Learning-Based Methods

Compared to other low-level vision tasks, the development of deep learning-based low-light image enhancement progressed relatively slowly until the advent of LL-Net in 2017 [34]. In 2018, Chen et al. [35] pioneered the development of a series of paired datasets with weak light input and normal exposure labels using long- and short-exposure shots, thereby propelling the advancement of deep network technologies for low-light image enhancement. Subsequently, methods based on deep learning gradually became mainstream.

The integration of Retinex theory with deep learning for low-light image enhancement was first proposed by Shen et al. [36]. They combined convolutional neural networks with Retinex theory, treating multiscale Retinex as a cascade of Gaussian convolutions with skip connections or in residual form, and designed a multiscale convolutional neural network, MSR-Net, based on paired data processed with Photoshop. The logarithmic transformation was used in the network to convert the Retinex model from a multiplication form to an addition form. However, this method tends to lose details due to the logarithmic transformation, which suppresses gradient changes in bright areas.

Subsequent works introduced Retinex theory into deep neural networks [11,18,35,37,38,39,40,41,42]. Among them, Retinex-Net [35] is the most inspiring method; it combines physical models and deep neural networks (DNNs). Following this, Zhang et al. proposed KinD [43] and KinD++ [38], offering more effective solutions. Unlike complex multi-stage training processes, Fu et al. [41] and Cai et al. [11] explored the possibility of end-to-end frameworks, achieving significant performance improvements.

According to the current literature statistics, almost one third of methods combine the design of deep networks with Retinex theory—for instance, designing different sub-networks to estimate the components of the Retinex model and estimating illumination maps to guide network learning. Although such combinations can integrate deep learning-based methods with traditional methods, their respective weaknesses may also be introduced into the final model: (1) the ideal assumption in Retinex-based low-light image enhancement methods, i.e., that reflectance is the final enhancement result, still affects the final outcome; (2) despite the use of Retinex theory, the risk of overfitting in deep networks still exists.

In cases where Retinex theory is not used, recent works have focused on directly sampling end-to-end methods [44,45,46,47,48,49,50,51] for low-light image enhancement. LLNet, proposed by Lore et al. [34], inspired the emergence of end-to-end methods, mainly showcasing the potential of supervised methods in enhancement. To mitigate color biases, some methods [52,53] employ three-dimensional look-up tables (3D-LUT) and histograms to maintain color consistency. In other methods [49,50], attempts have been made to use signal-to-noise ratio (SNR) perception priors and structure-aware features as guidance to produce realistic results. Recently, with the popularity of ultra-high-definition (UHD) images, methods such as LLformer, proposed by Wang et al. [12], and UHDFour, proposed by Li et al. [54], have been used to enhance UHD images, and related UHD datasets have been released to promote further research. Additionally, when training data are limited, semi-supervised [55,56], unsupervised [42,57], and zero-shot learning [58,59] methods are valuable research directions and important branches of deep learning-based low-light image enhancement.

## 3. Methodology

### 3.1. Separated Enhancement

**Figure 1:**
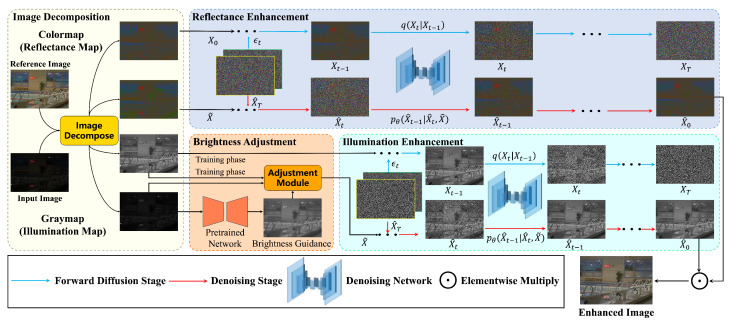
An overview of the framework. It contains four modules: image decomposition, brightness adjustment, reflectance restoration, and illumination restoration.

The proposed framework seeks to enhance the brightness of low-light images, drawing upon the foundational principles of Retinex theory [9], as shown in Figure 1. Central to this theoretical framework is the premise that low-light images can be systematically decomposed into two components: reflectance maps and illumination maps. The decomposition process can be formulated as follows:

(1) I=R∘L,

where ∘ designates the element-wise product, *I* represents the input low-light images, and *R* and *L* stand for the reflectance and illumination maps.

As indicated by [11], while a clean image is free from corruption, applying Retinex decomposition to low-light images results in reflectance and illumination maps that are marred by noise and artifacts, further complicating their estimation. Mathematically, a degraded low-light image can be naturally modeled as follows:

(2) $$I=\left(R+\hat{R}\right)\circ \left(L+\hat{L}\right),$$

where $\hat{R}$ and $\hat{L}$ are the polluted terms that perturbate the reflectance and illumination maps, respectively.

After the initial decomposition phase, the process of enhancing low-light images involves the application of element-wise multiplication using a ‘light-up map’ $\bar{L}$. This map is utilized to intensify the brightness of the input low-light image *I*. The mathematical formulation of this enhancement process can be expressed as follows:

(3) $$I\circ \bar{L}=\left(R+\hat{R}\right)\circ \left(L+\hat{L}\right)\circ \bar{L}=\left(R+\hat{R}\right)\circ \left(L\circ \bar{L}+\hat{L}\circ \bar{L}\right).$$

According to the above theory, decomposing the images in a Retinex manner consequently decouples the removal of the perturbation terms $\hat{R}$ and $\hat{L}\circ \bar{L}$ into two separate subspaces, allowing them to be better and more easily regularized/learned.

### 3.2. Retinex Decomposition Strategy

While we can recover low-light images by ensuring the better removal of perturbation terms in both the reflectance and luminance subspaces, how the Retinex is decomposed is still a fundamentally ill-posed problem, as indicated by a range of previous studies [28,35,43,60,61,62,63]. Some of them [28,61,62] have employed traditional methods to decompose low-light images into reflectance and illumination maps, which utilize channel-wise Gaussian blur to compute the illumination maps, subsequently obtaining the reflectance maps through the channel-wise division of the original image by the illumination maps. Meanwhile, learning-based methods typically involve the use of intricately designed deep neural networks (DNNs) to generate initial estimates of the reflectance and illumination maps, as seen in [35,38,43], often supplemented by hand-crafted constraints and priors [64,65,66,67,68,69], although these may be constrained by the model’s capacity.

Although these methods have marked significant advancements in the estimation of reflectance and illumination maps, achieving precise outcomes remains a challenge. Ref. [11] have demonstrated that while a clean image is free from corruption, the application of Retinex decomposition to low-light images results in reflectance and illumination maps that are marred by noise and artifacts, further complicating their estimation. Addressing this challenge, this study diverges from the pursuit of increasingly complex networks for more accurate initial estimations. Instead, we introduce a novel decomposition strategy that employs a fixed pattern, decomposing images into a graymap and colormap as the initial estimation of the reflectance and illumination maps, bearing similarity to [20,47], but with notable distinctions. This innovative strategy presents a unique paradigm for the problem of Retinex decomposition.

**Graymap**: We execute a weighted summation of the RGB channels, prioritizing the green (g) channel, followed by the red (r) and blue (b) channels, in descending order according to the weights set by the color space standards [70]. The formulation of this function is expressed as follows:

(4) L=0.299×r+0.587×g+0.114×b.

**Figure 2:**
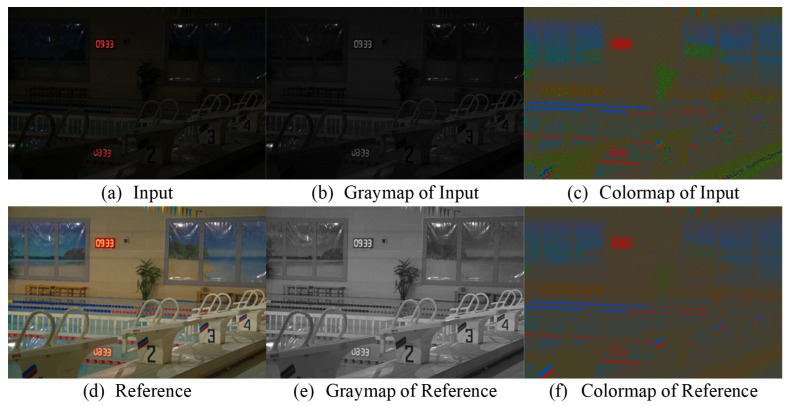
Examples of low-light and normal-light images, along with their corresponding colormaps and graymaps—which can be viewed as reflectance maps and illumination maps—are shown. The example image displayed is from the test set of the LOL dataset.

For the graymap, as illustrated in Figure 2b,e, our strategy deviates from the method in [47], which calculates the mean across the RGB channels. Recognizing the human eye’s heightened sensitivity to green, we assign greater weight to this channel. Notably, this weighting ensures that brighter pixels in the original image retain their prominence in the weighted sum, thereby preserving the strong correlation between the illumination map and the original image’s brightness. This characteristic renders the map suitable for use as the illumination component in the Retinex model.

**Colormap**: With the acquisition of the graymap, we can obtain the corresponding colormap by performing the pixel-wise division of each RGB channel by the graymap as follows:

(5) $$R=\left(\frac{r}{L},\frac{g}{L},\frac{b}{L}\right).$$

For the colormap, as illustrated in Figure 2c,f, the reflectance map exhibits a notable degree of consistency, even amidst varying luminance levels. This steadfast characteristic positions it as an apt candidate for the newly estimated reflectance map within the Retinex framework, leveraging its stability across different lighting conditions.

### 3.3. Conditional Diffusion Model

Conditional diffusion models are commonly used in image enhancement [13,71,72]. These models enhance images by inputting the degraded image as conditional information into a noise prediction network, guiding the diffusion model to generate an enhanced image corresponding to the degraded one.

Specifically, the forward process systematically introduces Gaussian noise into the clean image $X_{0}$. As delineated in [73], this Gaussian diffusion forward process incrementally contaminates the clean data $X_{0}$ through a sequential *T* diffusion time step mechanism, thereby enabling the acquisition of the sampled intermediate state $X_{t}$:

(6) $$q\left(X_{t}|X_{t-1}\right)=N\left(X_{t};\sqrt{\alpha_{t}}X_{t-1},\left(1-\alpha_{t}\right)\epsilon_{t}\right),\;\epsilon_{t}∼N\left(0,I\right).$$

where $\alpha_{t}=1-\beta_{t}$, $\beta_{t}$ represents the variance schedule, and $X_{t-1}$ is the intermediate state of the previous sampling steps. Additionally, $\epsilon_{t}$, drawn from a normal distribution N(0,1), possesses the same dimensionality as the input data $X_{0}$.

Given that ${\hat{\alpha }}_{t}=\prod_{i=1}^{t}\alpha_{i}$, the equation describing the aforementioned process can be simplified as follows:

(7) $$X_{t}=\sqrt{{\hat{\alpha }}_{t}}X_{t-1}+\sqrt{1-{\hat{\alpha }}_{t}}\epsilon_{t},\;\epsilon_{t}∼N\left(0,I\right).$$

The reverse process in diffusion models constitutes a denoising procedure, wherein the model is trained to effectively reconstruct a clean original signal from noisy data. Specifically, sampling is conducted using the Gaussian transitions $p_{\theta }\left({\hat{X}}_{t-1}|{\hat{X}}_{t},\tilde{X}\right)$, which are parameterized by learned parameters. This process initiates from ${\hat{X}}_{T}∼N\left(0,I\right)$ through the following mechanism:

(8) $${\hat{X}}_{t-1}=\frac{1}{\sqrt{\alpha_{t}}}\left({\hat{X}}_{t}-\frac{1-\alpha_{t}}{\sqrt{1-{\bar{\alpha }}_{t}}}\epsilon_{\theta }\left({\hat{X}}_{t},t,\tilde{X}\right)\right),$$

where ${\hat{X}}_{t}$ represents the sampled random Gaussian noise, ${\hat{X}}_{t-1}$ denotes the intermediate result following one step of the denoising process, and $\tilde{X}$ is the conditional guide parameter. In this study, we select the input low-light image to serve as the guide parameter.

Given its efficacy in addressing complex degradation patterns, this study utilizes a conditional diffusion model, specifically a typical patch-based conditional diffusion model [13], to effectively eliminate perturbation terms in the reflectance and illumination maps. The details of this framework are illustrated in Figure 1.

### 3.4. Brightness Adjustment Module

Given that the graymap effectively isolates color information, the primary objective is to enhance the brightness while concurrently eliminating perturbations. As depicted in Figure 2, the graymap of a low-light image typically exhibits a markedly low pixel intensity, leading to the significant loss of visible details. Consequently, it becomes imperative to augment the pixel values within the graymap. However, this enhancement process also tends to amplify the noise, thereby exacerbating the perturbations, and, consequently, the process of luminance enhancement can adversely impact the perturbation removal phase.

Previous research [20] has indicated that utilizing a low-light image directly as a conditional input does not produce optimal outcomes, underscoring the inherent challenges and inefficiencies in simultaneously achieving brightness enhancement and noise removal. To tackle this issue, we adopt a two-step approach, initially focusing on brightness adjustment, followed by the removal of perturbations. This methodology ensures that each aspect of image improvement is addressed effectively without adversely affecting the other.

**Brightness Guidance**: Recent studies [18,35,43] have often overlooked the complex interrelation between brightness enhancement and perturbation removal. Contrarily, our approach prioritizes brightness adjustment through brightness guidance. This strategy effectively elevates the brightness of low-light graymaps, albeit with a consequent increase in noise and artifacts. The subsequent use of a conditional diffusion model, therefore, concentrates exclusively on removing these noise and artifacts. This focused approach, by distinctly separating the tasks of brightness adjustment and disturbance removal, results in a more efficient enhancement process.

The utilization of brightness guidance is tailored differently for the training and inference phases. In the training phase, paired images enable the use of normal-light images for brightness reference. In contrast, the inference phase, lacking paired images, leverages a pre-trained network to adjust the low-light graymaps towards normal light, thus providing brightness guidance. The focus here is on matching the brightness levels, primarily using Gaussian-blurred reference graymaps, rather than preserving fine details. This approach ensures brightness alignment with the reference, making it an effective strategy for brightness guidance despite the potential limitations in detail retention.

The method for brightness adjustment comprises the following steps.

1. **Mean Gray Value Calculation**: Compute the mean gray value $g_{1}$ for blocks in low-light images and $g_{2}$ for blocks in normal light or as determined by the learned light model.
2. **Brightening Coefficient Determination**: Establish the brightening coefficient γ using the formula $\gamma =\frac{g_{2}}{g_{1}}$. This coefficient represents the factor for the enhancement of the brightness of the low-light graymap to produce the final conditional image. This approach ensures that the conditional image matches the guidance image in terms of the brightness level.

## 4. Experiments

We employ the Adam optimizer [74] to train the proposed diffusion model, with the parameter configurations as follows: the initial learning rate is set to $2\times 10^{-5}$, and no weight decay is applied. In the parameter updating process, exponential moving averages are utilized with a weight of 0.999 to promote more stable learning. For an RGB image, it is randomly cropped into image patches of size 64×64. Xavier’s method [75] is employed for the random initialization of the parameters in each module of the network.

We employ the PyTorch [76] deep learning framework to implement the training and testing processes of the neural networks. Throughout the network training, the total number of iterations is 960k, with the learning rate remaining constant throughout. Additionally, to expedite the sampling phase, the DDIM [77] training method is adopted, where the final augmentation results are obtained after every 15 iterations.

We train and evaluate the proposed model on the LOL [35] dataset, LOLv2-real [78] dataset, and LOLv2-syn dataset [78]. The LOLv2-real dataset comprises 689 pairs of low-/normal-luminance paired images collected from real scenarios, including 689 training pairs and 100 testing pairs, with an image resolution of 600×400. The LOLv2-syn dataset consists of 1000 pairs of synthetically generated low-/normal-luminance paired images, also with a resolution of 600×400. The batch processing approach is employed to feed the training data into the neural network, with each batch containing 16 pairs of samples. The entire experiment is conducted on an NVIDIA 1080Ti GTX GPU, and the training of the proposed network model takes approximately two days to fully converge.

## 5. Results

### 5.1. Comparison of Results on Real Datasets

**Figure 3:**
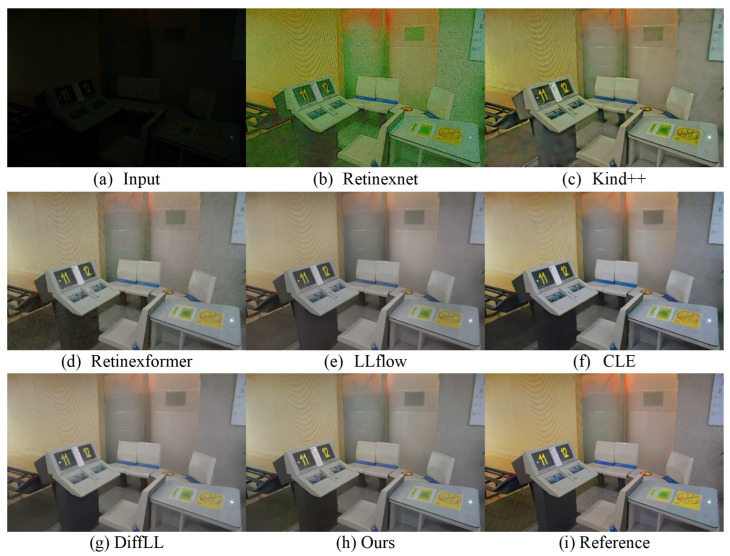
Visual comparison with other state-of-the-art methods on the LOL real-world dataset.

**Figure 4:**
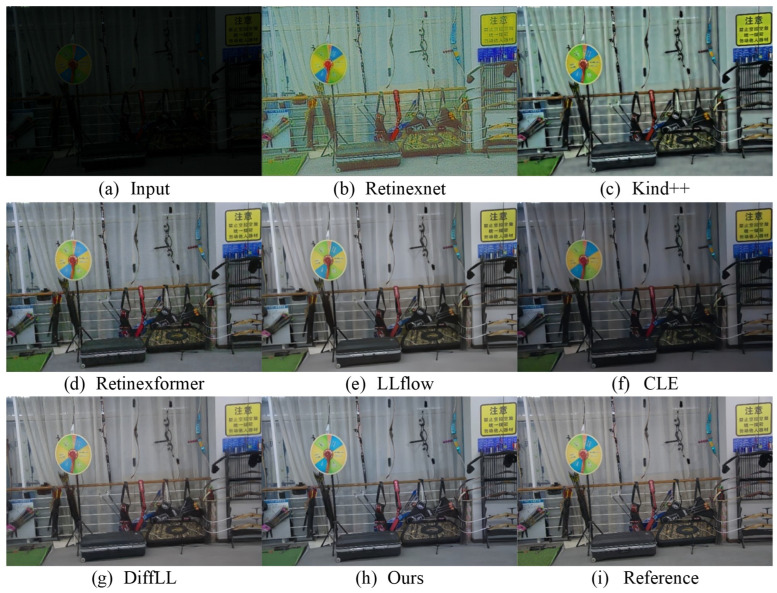
Visual comparison with other state-of-the-art methods on the LOLv2-real real-world dataset.

We conduct the testing of the proposed method on low-light images captured in real-world scenarios and visually compare its effectiveness with that of other algorithms. Figure 3 and Figure 4, respectively, depict the enhancement results of real-scene images captured from two different datasets, the LOL dataset and the LOLv2-real dataset. It is evident from the figures that the proposed method exhibits significant visual advantages over the state-of-the-art algorithms. Previous methods exhibit various shortcomings: the Retinexnet [35] method results in color distortion; the Retinexformer [11] method encounters difficulties in over-/underexposed regions and noise suppression; the DiffLL [15] and CLE [20] methods produce blurred areas; and the Kind++ [38] method introduces unnatural artifacts and edge distortions. In contrast, the proposed method has achieved significant success in enhancing the image clarity, as evidenced by the clearer visibility of the text in Figure 3. The sharpening of text edges and the enhancement in contrast have been effectively applied. In terms of brightness restoration, as shown in Figure 4, our method also demonstrates its advantages, effectively recovering the brightness information of the enhanced image to levels closer to the reference image. However, in terms of color restoration, although our method provides satisfactory results in most cases, it falls slightly short in terms of color accuracy and richness compared to the CLE method in certain specific scenes.

In order to better comprehend the effectiveness of the proposed method and other methods in enhancing real-scene images, various quantitative evaluation metrics, such as the peak signal-to-noise ratio (PSNR), structural similarity index (SSIM), and learned perceptual image patch similarity (LPIPS), were introduced. As depicted in Table 1 and Table 2, the quantitative metrics of the proposed method on both real datasets reached the state-of-the-art level.

### 5.2. Comparison of Results on Synthetic Datasets

**Figure 5:**
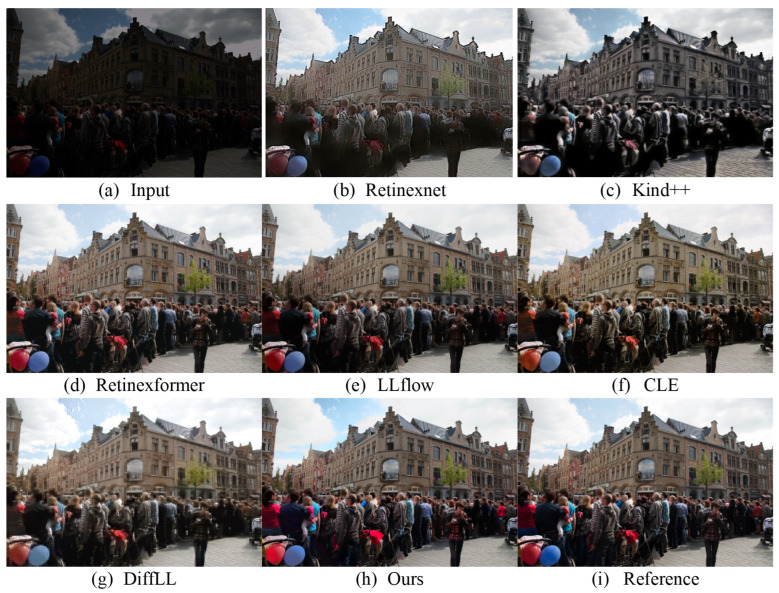
Visual comparison with other state-of-the-art methods on the LOLv2-syn synthetic dataset.

We utilize the LOLv2-syn dataset [78] to evaluate the enhancement efficacy of the proposed method and compare it with existing state-of-the-art algorithms. In the quantitative comparison of the results, we calculate the peak signal-to-noise ratio (PSNR), structural similarity index (SSIM), and learned perceptual image patch similarity (LPIPS) between the enhanced images and reference normal luminance images to quantitatively assess the enhancement quality of the methods. As depicted in Table 3, the proposed method exhibits higher objective evaluation metrics on the simulated dataset compared to the current state-of-the-art low-light image enhancement algorithms. Figure 5 displays a comparison of the enhancement results on an outdoor scene low-light image among various methods in the dataset. The proposed method effectively recovers the color fidelity of the image, rendering it more akin to that of a reference image, exemplified by elements such as the wall of a house. Additionally, the brightness levels and sharpness of the enhanced image align closely with those observed in the reference image, demonstrating the method’s capacity to maintain consistency in key visual parameters.

It is noteworthy that the benchmark model trained by the proposed method is based on a diffusion model. The performance improvement of the proposed method on the simulated dataset compared to the current diffusion models reaches 1.73 dB. Furthermore, on the previously tested real datasets, the improvement is also significant, reaching 1.15 dB and 0.3 dB, respectively. This indicates that the prior brightness information proposed by us contributes significantly to the low-light image enhancement task, showcasing promising enhancement.

### 5.3. Generalization Ability to Real-World Images

This section aims to elucidate the generalization performance of the proposed method by conducting a comparative analysis with current state-of-the-art methods. Training is conducted using the LOL training dataset, while testing is carried out on a variety of real-world low-light datasets captured in diverse scenes.

**Figure 6:**
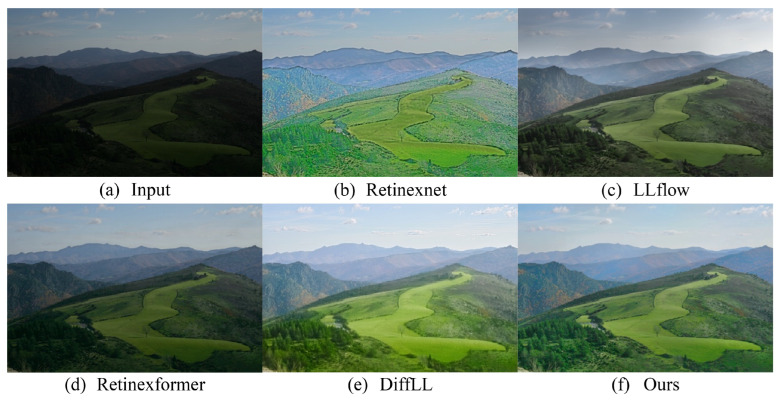
Visual comparison with other state-of-the-art methods on the MEF dataset.

**Figure 7:**
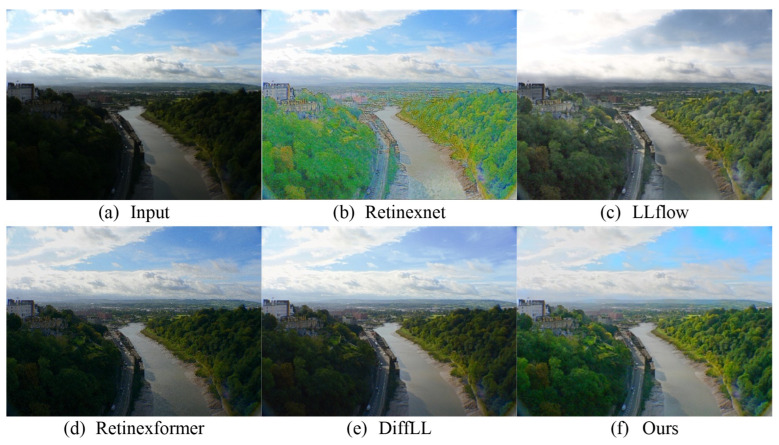
Visual comparison with other state-of-the-art methods on the VV dataset.

We conducted an extensive experimental analysis utilizing two classical low-light image datasets: MEF [79], which comprises 17 test images, and VV, containing 24 test images. The efficacy of our proposed method is illustrated through three sets of detailed visual comparisons in Figure 6 and Figure 7, which provide an intuitive assessment of the visual effects. The results reveal that our method effectively enhances dark regions while preserving the color fidelity. The outcome is visually pleasing, devoid of significant noise and color casts. In contrast, wang2022low and Retinexformer do not adequately enhance the image brightness, resulting in inferior visualization outcomes. On the other hand, while DiffLL produces visually appealing results, it sometimes suffers from localized overexposure or underexposure. These observations demonstrate that our method possesses robust generalization capabilities, delivering more naturalistic image quality in real-world scenarios.

**Figure 8:**
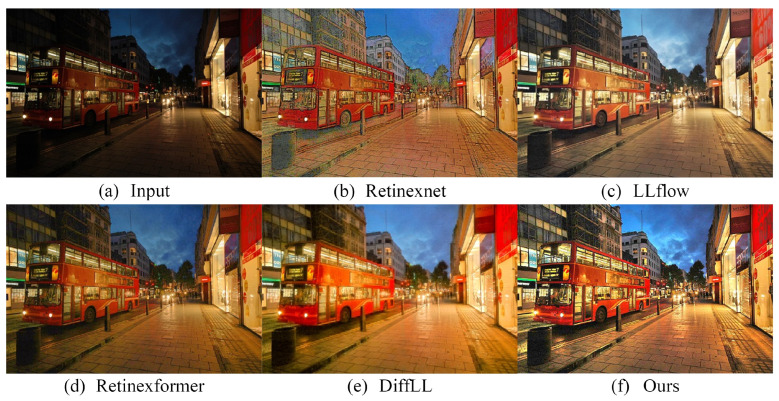
Visual comparison with other state-of-the-art methods on the ExDark dataset.

To further demonstrate the practical advantages of our method, we also performed experiments on a dataset designed specifically for object detection and recognition. For this purpose, we selected low-light images from the ExDark dataset [80] for testing. Figure 8 displays the comparative results, from which it is evident that Retinexnet is plagued by severe artifacts. wang2022low offers improved visualization, albeit with notable overexposure issues. DiffLL is marred by blurring effects in its results. Retinexformer, meanwhile, fails to adequately brighten the houses in the distance. Conversely, the results from our proposed method are visually superior, rendering the images more natural and clearer, particularly in areas such as the distant white houses and the sky.

The experimental results underscore not only the effectiveness of the proposed method but also the superior generalization performance of diffusion models compared to traditional convolutional neural networks (CNNs). As an emerging deep learning architecture, diffusion models excel in handling complex data distributions, offering significant improvements in areas like image processing. These findings highlight the potential of diffusion models to outperform traditional models in generalization capabilities, providing valuable insights for future research.

## 6. Ablation Study

This section will analyze and discuss the effectiveness of the novel Retinex decomposition strategy proposed in our method, followed by a discussion of the effectiveness of the prior brightness information proposed in our method.

### 6.1. Analysis of Differences in Image Decomposition Strategies

To rigorously evaluate our proposed Retinex decomposition strategy against traditional and contemporary methods, we conduct a series of comparative experiments. These experiments distinctly contrast our approach with the classical Single-Scale Retinex algorithm (SSR) [28] and the deep learning-based RetinexNet [35]. The aim is to validate our strategy’s effectiveness in enhancing the image quality and in detail preservation, highlighting its advancements over existing methods. The experimental conditions were standardized, except for the decomposition strategy, to ensure an accurate assessment of its efficacy.

The experimental results, as shown in Table 4, reveal the relative disadvantage of the traditional SSR method in the performance metrics. This disadvantage mainly stems from the inaccuracies of traditional decomposition methods in estimating the luminance component using Gaussian-blurred images. While Gaussian blur simplifies the representation of the image luminance, it often leads to the loss of important details, thereby affecting the naturalness and realism of the final image.

On the other hand, decomposition methods based on deep learning, although demonstrating comparable levels of learned perceptual image patch similarity (LPIPS, an index for the evaluation of the perceptual similarity between images) to our proposed approach, show a decline in performance in terms of the structural similarity index (SSIM, an index for the measurement of image quality) scores and peak signal-to-noise ratio (PSNR, an index reflecting the quality of image restoration). This outcome suggests that while deep learning-based methods are effective in handling some image issues, they may lead to the loss of image information in the encoder and decoder structures, particularly when attempting to separate delicate luminance information from the reflectance components, thereby revealing the limitations of such approaches.

**Figure 9:**
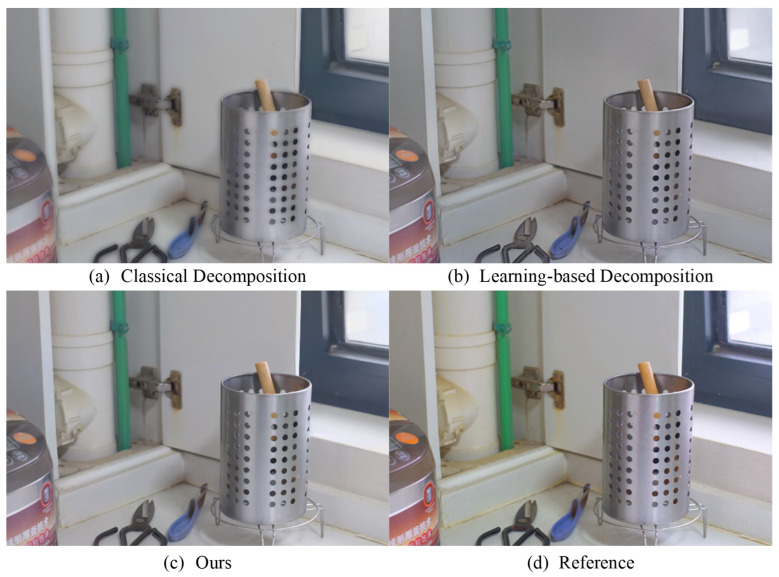
Comparison of enhancement results from different image decomposition strategies.

To further demonstrate the effectiveness of the proposed image decomposition strategy, the contrasting enhancement results of different decomposition strategies are provided. As illustrated in Figure 9, the traditional SSR-based decomposition strategy exhibits severe image blurring issues, while the deep learning-based decomposition strategy, although capable of enhancing images reasonably well, performs comparatively poorer in terms of color and saturation compared to the proposed decomposition strategy. Therefore, the proposed decomposition strategy ensures better performance in enhancing low-light images compared to existing decomposition strategies.

Our proposed image decomposition strategy is based on straightforward operations that separate image data into luminance and chrominance components. This simplicity leads to predictable and consistent outputs, which are particularly advantageous in scenarios where interpretability and reproducibility are critical. In contrast, learning-based methods, often reliant on complex neural networks, introduce a level of opacity due to their ‘black box’ nature. Moreover, because of its non-parametric nature, this decomposition strategy does not suffer from overfitting, a common issue in learning-based methods that can detrimentally impact their generalizability to new, unseen data. In conclusion, while learning-based decomposition methods continue to evolve and offer compelling benefits in certain applications, the simplicity, efficiency, and robustness of our proposed decomposition strategy make it an effective and reliable choice in many practical scenarios.

### 6.2. Analysis of Effectiveness of Prior Brightness Information

The application of prior brightness information contributes to enhancing the visual quality of images, particularly in processes involving brightness adjustment and disturbance removal. Prior brightness information not only guides enhancement algorithms to adjust the image brightness more accurately but also helps to maintain the naturalness and continuity of images when removing noise and disturbances.

To deeply understand the role of prior brightness information within the framework of the method proposed in our method, a comparative experiment was designed. By excluding the prior brightness information from the enhancement process, the impact of this change on the enhancement effect was observed. The experimental results, as shown in Table 5, indicate a significant decrease in the enhancement performance when the prior brightness information is not utilized. Specifically, the enhanced images exhibit noticeable inaccuracies and discontinuities in brightness, significantly compromising the visual quality of the images.

**Figure 10:**
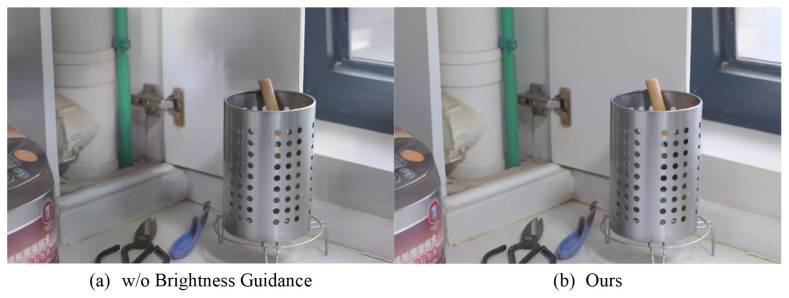
Comparison of enhancement results when removing prior brightness information.

Without prior brightness information, the tasks of brightness adjustment and disturbance removal have to be coupled together, making it difficult for the algorithm to balance the relationship between them, thereby affecting the final enhancement effect. As depicted in Figure 10, the brightness adjustment module struggles to accurately determine how to adjust the brightness of various regions in the image without guidance from prior brightness information, resulting in uneven brightness in the enhanced images. Similarly, disturbance removal becomes less effective due to the lack of prior brightness information, leading to the loss of image details or the generation of unnatural visual effects.

These comparative experimental results further emphasize the importance of prior brightness information in the image enhancement process. Prior brightness information not only helps to improve the accuracy of enhancement algorithms in brightness adjustment but also effectively guides disturbance removal, ensuring that the enhanced images maintain the natural brightness while enhancing the overall visual quality. Therefore, the guiding role of prior brightness information is crucial in ensuring fidelity and naturalness in the image enhancement process.

## 7. Conclusions

In this paper, we propose an adaptive brightness method. Utilizing a pretrained model, adaptive brightness information is extracted and mapped from low-light images, which is then enhanced through a brightness adjustment module. Subsequently, a conditional diffusion model is employed to mitigate the noise perturbations introduced by the brightness adjustment, thus separating the intertwined challenges of brightness enhancement and noise perturbation removal and reducing the complexity involved in enhancing the illumination component. Moreover, we introduce a simple yet effective image decomposition strategy that decomposes the image into graymaps and colormaps, serving as initial estimates similar to the illumination and reflectance components in Retinex decomposition.

To enhance the performance of low-light image enhancement, we employ diffusion models instead of convolutional neural networks to separately enhance the estimated illumination and reflectance components. For the reflectance component, due to its consistency under various lighting conditions, it is sufficient to directly use the conditional diffusion model to remove noise perturbations. In the comprehensive quantitative and qualitative analyses, our proposed method surpasses the current state-of-the-art across multiple datasets. Additionally, it demonstrates generalization capabilities through its performance on several real-world scenario datasets.

## Figures and Tables

**Table 1:** Quantitative comparison with other state-of-the-art methods on the LOL dataset.

Model Type	Method	PSNR↑	SSIM↑	LPIPS↓
CNN	Retinexnet [35]	16.77	0.539	0.474
	Kind++ [38]	21.80	0.876	0.158
Transformer	Retinexformer [11]	25.15	0.897	0.131
Normalizing Flow	wang2022low [47]	25.19	0.930	0.110
Diffusion Model	CLE [20]	25.51	0.888	0.164
	DiffLL [15]	26.32	0.898	0.118
	Ours	**27.47**	**0.929**	**0.098**

**Table 2:** Quantitative comparison with other state-of-the-art methods on the LOLv2-real dataset.

Model Type	Method	PSNR↑	SSIM↑	LPIPS↓
CNN	Retinexnet [35]	15.96	0.674	0.390
	Kind++ [38]	17.66	0.783	0.217
Transformer	Retinexformer [11]	22.79	0.866	0.171
Normalizing Flow	wang2022low [47]	25.42	0.892	0.157
Diffusion Model	CLE [20]	20.72	0.806	0.232
	DiffLL [15]	28.88	0.896	0.100
	Ours	**29.16**	**0.914**	**0.119**

**Table 3:** Quantitative comparison with other state-of-the-art methods on the LOLv2-syn dataset.

Model Type	Method	PSNR↑	SSIM↑	LPIPS↓
CNN	Retinexnet [35]	19.39	0.833	0.252
	Kind++ [38]	17.48	0.813	0.232
Transformer	Retinexformer [11]	25.67	0.952	0.059
Normalizing Flow	wang2022low [47]	26.06	0.957	0.047
Diffusion Model	CLE [20]	28.17	0.941	0.078
	DiffLL [15]	22.46	0.888	0.159
	Ours	**29.90**	**0.963**	**0.046**

**Table 4:** Ablation study on the decomposition strategy.

Decomposition Strategy	PSNR↑	SSIM↑	LPIPS↓
Classical Decomposition	24.58	0.850	0.171
Learning-Based Decomposition	23.69	0.912	**0.095**
Ours	**27.47**	**0.929**	0.098

**Table 5:** Ablation study on brightness guidance.

Prior	PSNR↑	SSIM↑	LPIPS↓
w/o Brightness Guidance	18.08	0.858	0.098
Ours	**27.47**	**0.929**	**0.098**

## Data Availability

The publicly archived datasets can be found at https://daooshee.github.io/BMVC2018website/, accessed on 17 April 2024 (LOL dataset) and https://github.com/flyywh/CVPR-2020-Semi-Low-Light, accessed on 17 April 2024 (LOL-v2 dataset).
